# Long-Term Outcomes of Patients with Biologically Treated Psoriatic Arthritis and Atopic Dermatitis—A Single-Center Experience

**DOI:** 10.3390/jpm14040427

**Published:** 2024-04-17

**Authors:** Georgiana Strugariu, Cristina Pomîrleanu, Mara Russu, Alexandra Popescu, Luiza Andreea Petrariu, Eugen Ancuta, Rodica Chirieac, Doinița Temelie-Olinici, Codrina Ancuța

**Affiliations:** 1 Department of Rheumatology, Faculty of Medicine, “Grigore T. Popa” University of Medicine and Pharmacy, 16 Universitatii Street, 700115 Iasi, Romania; georgiana-c-strugariu@d.umfiasi.ro (G.S.); chirica.mara@d.umfiasi.ro (M.R.); alexandra_popescu@email.umfiasi.ro (A.P.); codrina.ancuta@umfiasi.ro (C.A.); 2 Rheumatoloy 2 Department, Clinical Rehabilitation Hospital, 14 Pantelimon Halipa Street, 700661 Iasi, Romania; alpetrariu@yahoo.com; 3 Research Department, Elena Doamna Clinical Hospital, 700398 Iasi, Romania; 4 Sanocare Medical and Research Center, 700503 Iasi, Romania; chiriac01ro@yahoo.com; 5 Department of Morpho-Functional Sciences II, Faculty of Medicine, “Grigore T. Popa” University of Medicine and Pharmacy, 16 Universitatii Street, 700115 Iasi, Romania; doinita.p.olinici@umfiasi.ro

**Keywords:** psoriatic arthritis, psoriasis, atopic dermatitis, biologic therapy, TNF inhibitors, IL-17 inhibitors

## Abstract

(1) Background: Although the association between psoriasis and atopic dermatitis (AD) is reported in the literature, scarce data are known about the efficacy of biologic therapy (including TNF and IL-17 inhibitors) in patients with psoriatic arthritis (PsA) and concomitant AD. (2) Objective: We aimed to explore AD in patients with PsA undergoing biologics for their active disease, focusing on prevalence and clinical and potential therapeutic implications. (3) Material and methods: We performed a retrospective analysis of 64 patients with PsA receiving various biological agents, followed-up in an academic outpatient rheumatology department up to 10 years. (4) Results: Atopic diseases were reported in about one third of cases, with a higher incidence of AD (10 cases; 52.6%) vs. atopic rhinitis (6 cases; 31.6%) and allergic asthma (3 cases; 15.8%). Three morphological patterns of AD were recognized including chronic prurigo (3 cases), a chronic lichen simplex (1 case), and eczemas (6 cases). All PsA with concomitant AD displayed a late onset of skin atopy (in their adult life) and demonstrated a specific profile (younger), from urban settings, equally distributed among genders, and requiring switching to a higher number of biologics to achieve disease control. (5) Conclusion: PsA and AD may coexist, requiring special attention when selecting the optimal biologic agent.

## 1. Introduction

Spondyloarthritis (SpA)are heterogeneous chronic rheumatic immune–inflammatory disorders that include five major types: ankylosing spondylitis, psoriatic arthritis, arthritis associated with inflammatory bowel disease, reactive arthritis, and undifferentiated spondyloarthritis [1,2,3].

Psoriatic arthritis (PsA) is a specific form of peripheral SpA linked to the presence of cutaneous psoriatic disease and affects up to 30% of patients with psoriasis [4]; it is recognized as a systemic condition with a particular pathogenic chain involving immune cells implicated in the type 1 immune response, especially T helper (Th) 1 lymphocytes, Th17 and Th22, and their proinflammatory cytokine complex: tumor necrosis factor α (TNFα) and interleukins (IL–17, IL-22) [5,6]. Over the past 20 years, the treatment landscape of PsA has recognized a huge wave of therapeutic agents targeting proinflammatory cytokines [7] to control both musculoskeletal manifestations and skin psoriasis. Proinflammatory cytokines, such as TNFα or IL-17A, are specific for the type 1 aberrant immune response; thus, targeting these cytokines is an attractive way to stop or, at least, delay the pathogenic process. The same targets were considered for treatment of psoriasis.

Psoriasis is an inflammatory disorder that mainly affects the skin and involves Th1, Th17, and Th22 immune cells in the development and persistence of inflammation at the skin lesions [8]. The most common and well-known non-cutaneous manifestation of psoriasis remains PsA, while uveitis and inflammatory bowel disease are also reported [8]. 

The latest evidence suggests that immune disorders driven by opposing immune mechanisms, which usually suppress each other in healthy subjects, could coexist in the same patient [8,9,10,11]. Key insights into understanding the complex pathobiology of autoinflammatory and autoimmune rheumatic and non-rheumatic conditions have transcended the myth that type 1 immune response disease excludes type 2 immune disorders [9]. 

Atopy is the main immune system disorder in which type 2 inflammation plays a pivotal role [8]. It has been recognized that atopy is associated with immune–inflammatory rheumatic diseases [9,10,11] and this association is not altered by biological treatment [12]. Atopic disorders, which include atopic dermatitis (AD), allergic asthma (AA), and hay fever or atopic rhinitis (AR), are disorders known as type 2-dominated immune responses, where Th2 is the primary ruler along with eosinophils, mastocytes, and lymphocytes B [13]. Atopy first develops in childhood, and usually begins with atopic dermatitis, while, later in life, patients might develop other forms of atopy. Atopic dermatitis is defined as a chronic, itchy inflammatory disease of the skin; being commonly reported in young age groups, atopic dermatitis could also present an onset in adults (over 18 years) or, even, an elderly onset (over 65 years) and could, therefore, be considered as a lifelong disease [13,14]. AD is broadly characterized by different clinics, depending on the age of onset; there are six recognized major clinical patterns: lichenoid atopic dermatitis, juvenile plantar dermatosis, nummular-type atopic dermatitis, follicular atopic dermatitis, eczema coxackium, and psoriasiform atopic dermatitis [8].

Psoriasis and atopic dermatitis are two of the most common inflammatory skin diseases, with a prevalence of 3–10% [8,15], still increasing due to multiple external factors targeting the skin [8]. However, the co-occurrence of both diseases in the same individual is less common than expected based on their prevalence in the population [14,15,16]. It is reported that AD has a higher prevalence in the global population than psoriasis especially in young patients, but their prevalence seems to be equal in adulthood, and the coexistence of AD–psoriasis was found in 1.3% patients [8]. Despite numerous reports of the coexistence of psoriasis and AD, there are no published data on the association of AD with PsA. 

Both psoriasis and atopic dermatitis are driven by opposing immunological mechanisms [17], which appear to be hard to control therapeutically when they coexist. The implication of Th17 and Th22 in acute and chronic phases of inflammation in AD and psoriasis [18,19,20], and their implication in etiopathogenesis of PsA [6], creates the molecular context of the coexistence of these three diseases in rare cases, and such a complex immune profile could help guide the biological therapeutic approach for these patients.

Although biological therapies such as TNF inhibitors and IL-17A inhibitors are already widely used for the treatment of psoriasis and PsA over the past two decades, only very little is known about their effects on the entire immune system long-term. Moreover, up to 30–40% of biologically treated patients do not achieve the optimal therapeutic goal under TNF inhibitors, while the biological basis of non-responders is still unknown [1]. 

Recent studies have already proved that anti-TNFs in patients with SpA are able to alter the innate immune system, but not the Th1/Th17 immune network [1]. Furthermore, long-term use of TNF inhibitors has been correlated with either the development or worsening of clinical manifestations of various forms of atopy [12]. Given this, for many rheumatologists, choosing the optimal biological molecule as the first treatment line has been difficult, especially with many available molecules. Identifying a specific profile of a patient in order to personalize the biological therapy is, therefore, essential to treat and target without switching or swapping biologics. Atopy could be helpful for this personalized therapy. 

It appears that the prevalence of atopic disorders in patients with autoimmune and inflammatory rheumatic and non-rheumatic conditions is scarce; to our knowledge, there are only limited data on the association or coexistence of atopy with psoriasis and PsA, particularly for those patients undergoing biological therapy for PsA. It is currently unknown whether PsA with and without atopy, especially AD, will respond differently to biologics as well as to targeted synthetic disease-modifying anti-rheumatic drugs (tsDMARDs), such as Janus kinase (JAK) inhibitors; however, the intervention of specific proinflammatory cytokines (TNF α vs. IL-17 axis vs. IL-12/23 axis) in patients with AD, psoriasis, and PsA is able to promote different effects in response to different biologics, raising the question that AD might be a factor guiding biologic drug selection in patients with PsA and atopy.

In order to validate our hypothesis suggesting an individualized therapeutic response to biological therapy in patients with psoriatic arthritis and concomitant atopy, we explored the frequency of clinical manifestations of atopic dermatitis among patients with psoriasis and PsA under biological treatment with the aim of identifying potential clinical and therapeutic implications.

## 2. Materials and Methods

We conducted a retrospective analysis of 64 consecutive patients fulfilling 2006 CASPAR criteria for the classification of PsA, who received biologic therapies (TNF inhibitors, IL-17 inhibitors) for their active disease and underwent at least one efficacy assessment after six months of therapy. 

**Figure 1:**
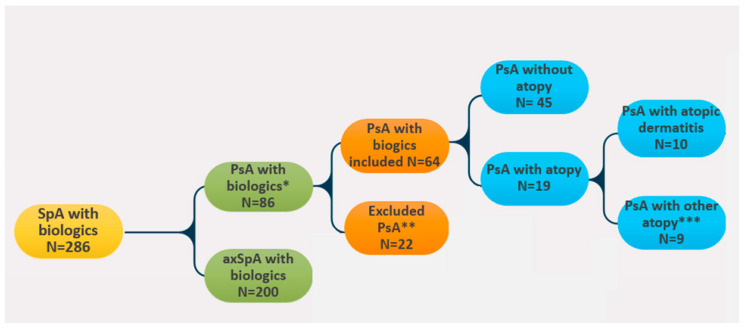
Flow-chart of patients with PsA enrolled; * diagnosed according to 2006 CASPAR criteria; ** loss of evidence or declined participation in study; *** patients with concomitant allergic asthma and allergic rhinitis.

Patients were selected from a large cohort of 286 patients with SpA who were initiated on biologics and had regular follow-ups in our academic center of rheumatology between January 2013 and December 2023; among them, 200 patients had axial SpA and were excluded from the beginning, 86 had a diagnosis of PsA according to the CASPAR criteria, and only 64 patients with PsA were included in our study as they had active follow-up visits in our department during the study enrollment phase (January–December 2023). Enrolled patients were classified in two subgroups, with and without concomitant atopic disorders (atopic dermatitis, allergic asthma, allergic rhinitis). The flow-chart presenting the patient selection process and stratification based on the presence of atopy is shown in Figure 1.

According to local guidelines, biological therapy has been fully reimbursed for the management of active PsA since 2002; all five TNF inhibitors (TNFi) including original and biosimilars of infliximab, original and biosimilars of adalimumab, original and biosimilars of etanercept, golimumab, and certolizumab pegol received governmental endorsement; more recently (2017), that has extended to IL-17A inhibitor (IL-17i) secukinumab; ixekizumab since 2021; and the IL-23 inhibitor guselkumab since 2023. 

Patient candidates with PsA for reimbursed bDMARDs required three concomitant criteria: (1) a definite diagnosis of PsA according to the 2006 Classification Criteria for Psoriatic Arthritis (CASPAR); (2) severe PsA with high disease activity as defined by a Disease Activity in Psoriatic Arthritis (DAPSA) score > 28 despite conventional synthetic drugs (csDMARDs); (3) failure of at least two csDMARDs, meaning persistent active disease after 12 weeks with the maximum recommended doses, except for patients with predominantly axial PsA and those with active enthesitis and/or dactylitis, in which the use of NSAIDs at the maximum doses in the past 12 weeks is appropriate. We included only patients treated with TNFi and non-TNFi, secukinumab, since the last two approved agents, ixekizumab and guselkumab, have a shorter time of exposure compared with the other biologics.

For all patients we collected (i) demographic data; (ii) disease-related parameters including disease subtype (axial, peripheral), disease duration, individual articular and cutaneous (extension and severity of psoriasis PASI) parameters, and disease activity scores such as Disease Activity in Psoriatic Arthritis (DAPSA); and (iii) drug-related parameters including the class of bDMARDs, persistence on a specific drug, and the number of switched bDMARDs.

As a part of the enrollment process, all patients were interrogated about the history of atopic disorders: allergic asthma, allergic rhinitis, and atopic dermatitis. They were questioned about the moment of diagnosis of atopic disorders in relation to the evolution and diagnosis of psoriasis and PsA. A diagnosis of AA and AR was accepted if the patient had already been evaluated by a dermatologist who decided on a positive diagnosis of AA and AR and recommended treatment. Those who reported manifestations equivalent to atopic skin without a positive diagnosis were referred to a dermatologist for the confirmation of atopic dermatitis. The diagnosis of AD was established according to international guideline criteria of UK Working Group and American Academy of Dermatology for AD in both pediatric and adult populations [16]; the nomenclature of the different morphological phenotypes was used according to the International Classification of Diseases, Tenth Revision (ICD-10/L20.x) system [16]. 

We divided the patients with PsA and associated atopy into two groups: patients with atopic disease other than atopic dermatitis (non-AD PsA) and patients with concomitant PsA and atopic dermatitis (PsA-AD). We were mainly interested in analyzing those patients with PsA and associated atopy.

A statistical analysis was performed using the OPENSTAT (William G. Miller, Ames, IA, USA) software; all variables had a non-parametric distribution, as demonstrated by Shapiro–Wilk and Lilliefors tests; Mann–Whitney and chi-squared tests were used in the subgroup analysis (PsA with and without atopy), and a statistically significant *p*-value was considered if <0.05. 

## 3. Results

### 3.1. Psoriatic Arthritis under Biologics: General Findings 

Demographic, clinical profile, and therapeutic data of patients with PsA included in the current study are summarized in Table 1. We performed an overall analysis as well as a subgroup analysis in patients with various atopic conditions. 

A total of 64 patients with biologically treated PsA with active follow-up in our database were eligible for this study. The majority of cases were male (57.8%), and there was a mean age of 57 ± 6.8 years, mean duration of PsA of 16.22 ± 9.18 years, and mainly peripheral disease subtype with polyarticular involvement (94%). 

Most patients received at least one TNF inhibitor (57 patients, 89%), while 11 (17%) received IL-17 inhibitors either as a first-line biologic agent (7 patients) or at switching (4 patients). The mean exposure to biologics was 8.28 ± 4.61 years for TNF inhibitors and 0.56 ± 0.5 years for IL-17 inhibitors, respectively.

### 3.2. Psoriatic Arthritis and Concomitant Atopy 

**Figure 2:**
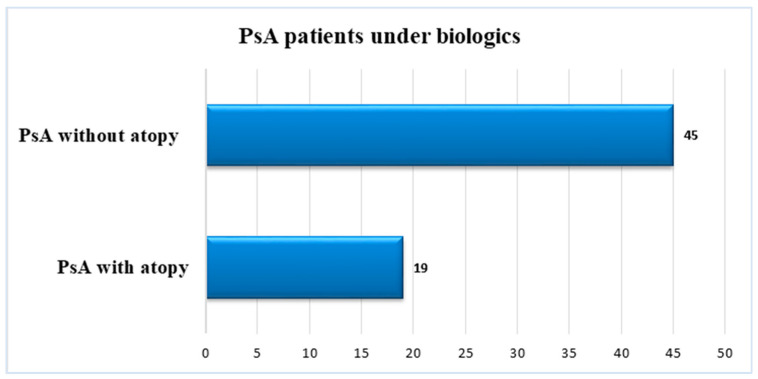
Patients with and without atopy in our cohort of PsA.

**Figure 3:**
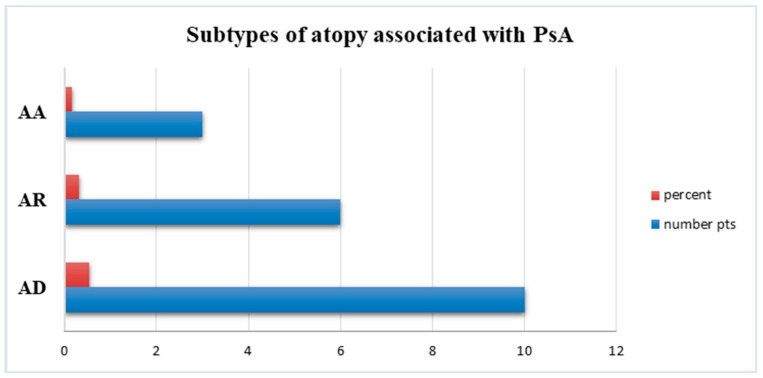
Distribution of atopic disorders in PsA with atopy subgroup; AD, atopic dermatitis; AR, allergic rhinitis; AA, allergic asthma; pts, patients.

A total of 45 patients (70.4%) were negative for atopic diseases, while 19 (29.6%) of 64 patients with PsA reported a history of or concomitant clinical manifestations of certain subtypes of atopy (Figure 2) with the following distribution: 10 cases (52.8%) with atopic dermatitis, 6 patients (32%) with allergic rhinitis, and 3 cases (15.8%) with allergic asthma (Figure 3).

**Figure 4:**
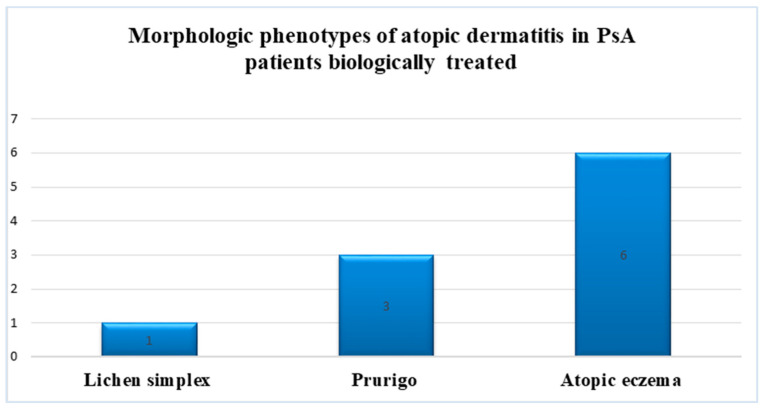
Morphologic phenotypes of atopic dermatitis in patients with PsA biologically treated.

Diagnosed by the presence of pruritus and rashes compatible with eczema without other extrinsic allergic triggers for allergy (chemicals, drugs, cosmetics, or food), atopic dermatitis was demonstrated in 15.6% of all cases (10 patients) of all patients with biologically treated PsA: three patients experienced chronic prurigo, one case presented a chronic simplex lichen, and six cases presented with various atopic eczemas (morphological subtypes: flexural, erythematous, or other atopic dermatitis) (Figure 4). 

A questionnaire about atopic disorders was conducted during the last checkup in our department. All ten patients declared the presence of atopic dermatitis at the time of the diagnosis of PsA concomitantly with psoriasis, and only twelve patients declared a long history of atopy in adult life, starting before the diagnosis of PsA (one, AA; two, AR; nine, AD). All reported persistent atopy while taking disease-modifying drugs, conventional synthetic drugs, or TNF inhibitors. 

All cases were associated with active skin psoriasis, although with a PASI score (Psoriatic Area and Severity Index) of less than 10%. The patient with a chronic simplex lichen reported a slight worsening of the skin lesion during infliximab treatment over the past 11 years; however, a good therapeutic response to infliximab has been demonstrated according to EULAR-ACR guidelines [21]. None of the cases of atopic dermatitis required treatment other than the regular intermittent systemic antihistamines or topic ointments. Based on local regulations, reimbursement of targeted molecules (Janus kinase inhibitors) for treatment of atopic dermatitis is available from 2023, but none of the patients in our cohort were eligible for this treatment according to the national protocol for atopic dermatitis.

### 3.3. Psoriatic Arthritis with and without Atopy—Comparative Data

We further explored demographics, disease-related parameters, and targeted treatments in patients with PsA and without atopy vs. the PsA with atopy subgroup, as shown in Table 1.

#### 3.3.1. Demographics 

There were no major gender differences in PsA, although male patients were slightly in favor in the overall group; however, the male-to-female ratio was 1:1 in all atopic groups with or without atopic skin disease (atopic dermatitis).

Atopy was reported to be more common in patients from urban communities, regardless of atopic disorder subtype: 15 patients (79%) out of 19 PsA cases with concomitant atopy, 8 patients (42%) with non-cutaneous atopy, and 7 patients (37%) with atopic dermatitis. In the overall group, the incidence of patients in the urban areas was 59.4% (38 patients out of 64). 

Patients with PsA and atopy were younger than those in the general group. Although our cohort has a mean age of 57 ± 6.8 years with 11% (seven patients) under 40 years old, the atopic group has an average age of 51 ± 6.5 years, 31.5% (six patients) being younger than 40 years old. Patients with non-cutaneous atopy appear to be younger, with an average age of 40 ± 3.4 years, and with the highest percentage of patients under 40 years of age (33.33%) of all groups. In the AD group, 80% were over 40 years of age and all patients reported adult-onset AD. 

#### 3.3.2. Disease-Related Parameters 

The peripheral PsA subtype (polyarthritis, enthesitis, dactylitis) was the dominant clinical pattern regardless of concomitant atopy, while the axial subtype was described in some cases. 

The mean duration of the evolution of PsA in the main cohort was 16 ± 9.12 years, and in the AD group, it was 13.85 ± 9.9, similar to the rest of the atopic groups. There was a delay of 3.1 ± 6 years between the PsA onset and diagnosis in all patients regardless the groups. 

#### 3.3.3. Treatment-Related Parameters

The mean treatment persistence was 8.28 ± 4.61 years for TNF inhibitors, and only 0.56 ± 0.56 years for IL-17 inhibitors. Of the 57 patients who underwent TNF inhibitors, only 16 patients (28%) remained on the same molecule for up to ten years. In addition, 41 (71.9%) patients with PsA required multiple switching occurrences between several TNF inhibitors to achieve or maintain the treatment target. The most widely used biologic drugs in 57 patients were iTNF, as it was the first class approved and widely used; only 11 patients had iIL-17 use between 2017 and 2023. The median follow-up time in our study was 6.68 ± 3.5 years. 

**Figure 5:**
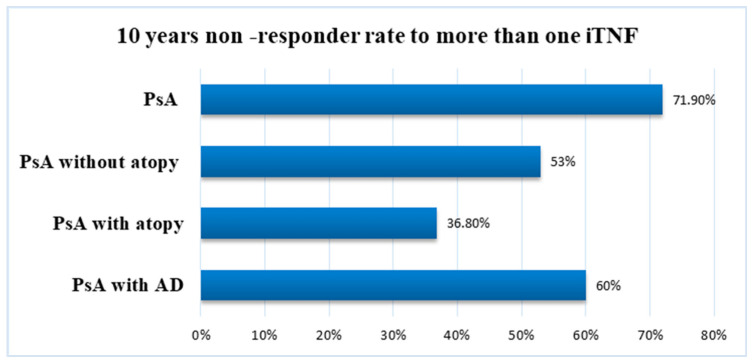
Non-responder rate between 2013 and 2023 to more than one iTNF in our study groups.

In the atopic group, 12 (63%) patients were treated with TNF inhibitors, and 7 (36.8%) required more than one TNF inhibitor for an optimal control of PsA. In the AD group, six patients were treated with at least two (range: 2–4) biologics; four patients were treated with secukinumab as the first line within the past four years (Figure 5). 

Data are not statistically significant because the groups were too small and the number of patients treated as well as the exposure time were different depending on the therapeutic class. In addition, the results also reflect that the atopic group and its subgroups are homogeneous and the biologics did not change the parameters tracked. However, the following are some observations that we can draw from these results: atopy is common in young patients with PsA, usually with an urban lifestyle, and the female/male ratio in the atopic group is 1:1.

## 4. Discussion

Although the association between psoriasis and atopic dermatitis, two immune-mediated chronic inflammatory skin diseases with specific pathobiological pathways, is not uncommon [18,22], data on concomitant psoriatic arthritis and atopic dermatitis as well as the impact of biological treatments (including TNF and IL-17A inhibitors) are lacking. The application of the last evidence and guidelines for the management of patients with PsA indicates that comorbidities and associated conditions may affect the choice of biologic agent, promoting personalized treatment plans for these patients [23]. Therefore, the association of psoriasis—psoriatic arthritis—atopic dermatitis remains particularly challenging as it could have a major role in selecting the optimal drug to target both skin conditions and the joint involvement. 

We focused on 64 consecutive biologically treated patients with PsA (both bio-naïve and bio-experienced) followed-up in our academic rheumatology department over 10 years; these patients were retrieved from a cohort of 286 patients with various biologically treated spondyloarthropathies in our database, receiving different biologics according to local recommendations. 

We analyzed the relation between the atopy and TNF inhibitors as well as IL-17 inhibitor secukinumab administered for active psoriatic disease. We were able to confirm that atopy is not altered by long-term treatment with iTNF, and AD associated with psoriatic lesions does not respond in the same way to various anti-TNF agents. Indeed, current treatment options for AD include different classes of drugs (topical medication, systemic agents, immunosuppressive agents, and biologics) aiming at controlling the symptoms of atopy; however, newer and innovative drugs, including targeting synthetic DMARDs such as JAKinibs (baricitinib, tofacitinib, upadacitinib), are undoubtedly facilitating optimal outcomes for both skin conditions and joint disease [24,25,26,27]. To our knowledge, this is the only study exploring the frequency of the association of atopic disorders (especially atopic dermatitis) with PsA and the potential outcomes of biologic treatments.

We noticed that atopy did not change during the i-TNFs; in addition, in some patients, various symptoms of atopy might interfere with routine monitoring of biologic effectiveness in clinical practice. Atopy has a wavy course, not parallel with PsA, and is influenced by external factors. Atopy is not associated with a better response to biologics, and the rate of non-responders in these cases is higher than patients with SpA in general or patients with PsA [12]. More than 30% of patients biologically treated for rheumatic immuno-inflammatory diseases do not respond to biologics after the first year [1]. In our PsA cohort, the rate of non-responders to one biologic reached 71.9% in 10 years of follow-up and 36.8% in the atopic group and more than half of the patients in the AD group required more than two molecules to treat and target according to national and international recommendations for the management of patients with PsA. 

Indeed, our results were not statistically significant (*p* > 0.05); one potential explanation is the small number of patients in the atopic group and its subgroups (atopic dermatitis and other atopic conditions). Nevertheless, we show that there is an association between PsA and atopic disorders in 29.6% of patients, unaltered by the biological treatment, especially by the iTNF. In addition, these patients usually required multiple molecules in 36.8% of cases in order to achieve an optimal control of PsA [21]. We need a better control by keeping the non-responder rate as low as possible, given the price that comes with multiple and more expensive therapeutic switches and the possible impact on the immune system of several biologics, which is not yet completely understood. We can achieve this by identifying clinical aspects, such as comorbidities, to narrow down the possible therapeutic molecules best suited to achieve the therapeutic target in maximum safety conditions with the molecule(s) and to maintain the optimal response as long as possible. 

Janus kinase inhibitors (iJAKs) are the novel therapeutic molecules that block multiple proinflammatory and pro-atopy cytokines. Their beneficial therapeutic role in treating atopic dermatitis and asthma is already known [26,27,28]. iJAK has received approval for treatment of psoriatic skin disease and psoriatic arthritis in the past 2 years in our country, increasing the number of the DMARDs and making it more difficult to choose from the first line to the most beneficial therapeutic molecule. It is expected that, in patients with PsA, the possible association with atopic diseases will be considered when it is time to choose a new DMARD line, as it is reported in the literature that iTNF does not change the course of atopy in patients with SpA and is linked to a higher rate of non-responsiveness [12]. 

Obviously, atopy is not listed among the common comorbidities for patients with PsA that mainly encompass metabolic syndrome entities (hypertension, hypercholesterolemia, diabetes, obesity, smoking, myocardial infarction), psychiatric conditions such as depression and anxiety, and osteoporosis [29,30]. But it can only be a new factor guiding the therapeutic response and, consequently, can be used to optimize therapeutic decisions.

We consider it of interest to further extend the assessment in larger cohorts of patients with PsA under biologics, aiming to prospectively evaluate the therapeutic response using atopic dermatitis as a criterion for the selection of an appropriate biologic agent or JAK inhibitor. If our data could be replicated in multiple clinical settings, it will be of real help to incorporate cross-disciplinary and collaborative approaches to actively coordinate management for patients with PsA. 

## 5. Conclusions

We raised the question of whether associated atopy in patients with PsA is a useful clue to identify an important profile of patients responding to a specific treatment. Screening patients with psoriasis and PsA for atopy and for AD could play an important role in creating a more specific profile of respondents to a particular therapeutic class and give these patients the chance for better control and a longer therapeutic response.

## Figures and Tables

**Table 1:** Demographic-, disease-, and treatment-related parameters in PsA cohort.

Parameters	PsA lot 64 pts *n* (%)	Atopic PsA ^1^ 19 pts (29.6%) *n* (%)	Non-AD PsA ^2^ 9 pts (14%) *n* (%)	PsA-AD ^3^ 10 pts (15.6%) *n* (%)	*p* * < 0.05 (Chi-Square)
Gender, *n* (%)					
Expected total/chi-square.					
Male	37 (57.8)	9 (10.25) [0.15]	4 (4.85) [0.15]	5 (5.39) [0.03]	
Female	27 (42.2)	10 (8.75) [0.18]	5 (4.15) [0.18]	5 (4.61) [0.03]	0.77 (1.1056)
Living style, *n* (%)					
Expected total/chi-square.					
Urban	38 (59.4)	15 (12.67) [0.43]	8 (6.00) [0.67]	7 (6.67) [0.02]	
Rural	26 (40.6)	4 (6.33) [0.86]	1 (3.00) [1.33]	3 (3.33) [0.03]	0.18 (4.8707)
Age (years), *n* (%)					
Expected total/chi-square.					
<40	7 (11)	6 (3.54) [1.71]	3 (1.68) [1.04]	3 (1.86) [0.69]	
41–60	28 (44)	7 (7.82) [0.09]	2 (3.71) [0.79]	5 (4.12) [0.19]	
>60	29 (45)	6 (7.64) [0.35]	4 (3.62) [0.04]	2(4.02) [1.01]	
Mean age (years)	57 ± 6.8	51 ± 6.5	40 ± 3.4	50 ± 3.7	0.20 (8.4688).
Skeletal involvement, *n* (%)					
Expected total/chi-square.					
Axial	17 (26.5)	7 (4.99) [0.81]	4 (2.36) [1.13]	4 (2.36) [1.13]	0.33 (3.3982)
Peripheral	60 (94)	12(14.01) [0.29]	5 (6.64) [0.40]	5 (6.64) [0.40]	
Duration of PsA **					
Mean ±SD	16.22 ± 9.18	13.5 ± 9.3	13.4 ± 8.7	13.85 ± 9.9	
Median (IQR)	14 (40) 0.95	14 (37) 0.93	13 (37) 0.97	10 (35) 0.98	0.98
Years from onset to diagnosis **					
Mean ± SD	3.11 ± 6.15	3.12 ± 6.13	3.11 ± 6.2	3.8 ± 6.12	
Median (range)	0 (33) 0.98	0 (27) 0.96	0 (27) 0.97	0 (33) 0.94	0.96
Years of PsA diagnosis **					
Mean ± SD	13.46 ± 8.97	10.4 ± 6.9	10 ± 5.6	10.5 ± 7.5	
Median (range)	0 (40) 0.8	0 (37) 0.98	0 (33) 0.97	7 (40) 0.88	0.97
Years on iTNF **					1
Mean ± SD	8.28 ± 4.61	8.15 ± 3.88	8.1 ± 3.6	7.25 ± 3.25	
Median (range)	8 (17) 1	8 (17) 1	8 (16) 1	7 (15) 1	
Years on iIL-17 **					
Mean ± SD	0.56 ± 0.56	0.55 ± 0.53	0.54 ± 0.47	0.58 ± 0.49	
Median (range)	0 (5) 1	0 (5) 1	0 (5) 1	0 (4) 1	1

* *p* calculated with chi-square calculator online: https://www.socscistatistics.com/tests/chisquare (accessed on 12 January 2023); ** parameters expressed by the mean, standard deviation (SD), and median with range calculated with OPENSTAT programs, and Shapiro–Wilk, Lilliefors, and Mann–Whitney tests. ^1^ Atopic PsA—group with PSA associated with atopic disorders. ^2^ PsA–non-atopic dermatitis—group with PSA associated with atopic disorders other than AD: AR or AA. ^3^ PsA–atopic dermatitis—group with PSA associated with atopic dermatitis.

## Data Availability

The original contributions presented in the study are included in the article, further inquiries can be directed to the corresponding author/s.
